# Bond–Slip Behavior of High-Strength Stainless Steel Wire Mesh in Engineered Cementitious Composites: Numerical and Theoretical Analysis

**DOI:** 10.3390/ma17235700

**Published:** 2024-11-21

**Authors:** Xuyan Zou, Tao Zhang, Ziyuan Li, Juntao Zhu, Ke Li, Minghao Peng

**Affiliations:** 1 Department of Civil Engineering, Zhengzhou University, Zhengzhou 450001, China; 2 Department of Civil Engineering, Zhengzhou Institute of Technology, Zhengzhou 450044, China

**Keywords:** high-strength stainless steel wire mesh, engineered cementitious composites, local bond–slip model, finite element analysis

## Abstract

This study introduces high-strength non-prestressed steel strands as reinforcement materials into Engineered Cementitious Composites (ECCs) and developed a novel high-strength stainless-steel-strand-mesh (HSSWM)-reinforced ECC with enhanced toughness and corrosion resistance. The bonding performance between HSSWM and an ECC is essential for facilitating effective cooperative behavior. The bond behavior between the HSSWM and ECC was investigated through theoretical analysis. A local bond–slip model was proposed based on the average bond–slip model for HSSWM and ECCs. The results indicated that the local bond–slip model provided a more accurate analysis of the bonding performance between HSSWM and the ECC compared to the average bond–slip model. The effects of the ECC’s tensile strength, steel strand diameter, and transverse strand spacing on local bond–slip mechanical behavior were investigated through FEA. The results showed that the local bond–slip model and FE results aligned well with the experimental data. Additionally, the distribution of bond stress between the HSSWM and the ECC was analyzed using the micro-element method based on the local bond–slip model. A prediction model for the critical anchorage length and bond capacity of HSSWM in the ECC was established, and the accuracy of the model was verified.

## 1. Introduction

ECCs exhibit tensile strain-hardening and multiple cracking characteristics due to the bridging effect of the fibers compared with traditional concrete [1]. However, existing research has shown that the improvement in bearing capacity of structural members is limited when strengthened solely with ECCs, primarily due to the restricted tensile strength of ECCs. Therefore, ECCs need to be combined with reinforcing materials to achieve greater performance improvements.

Cooperative behavior relies fundamentally on the bonding performance between the reinforcing materials and the matrix. To enhance the mechanical properties of ECCs, researchers have conducted extensive studies on their bonding performance with reinforcing materials. Martí et al. [2] analyzed the behavior of prestressed steel strands in concrete with different embedment lengths. A functional relationship between the average bond stress and the compressive strength of concrete was established, and formulas for calculating the transfer length and anchorage length of prestressed strands were provided. Shan et al. [3] studied the bond–slip behavior between ECCs and deformed steel bars through pull-out tests and acoustic emission monitoring, systematically elucidating the damage characteristics and bonding performance degradation of ECC–steel bars. Xu et al. [4] analyzed the distribution of bond stress and relative slip along the anchorage length between ultra-high toughness cementitious composites and steel bars, and they established a bond–slip constitutive model for steel bars and ECCs that accounts for anchorage position. Mi et al. [5] conducted experimental and theoretical analyses on the bonding performance between FRP bars and ECCs. A bond–slip constitutive relationship considering the influence of anchorage location on the interaction between FRP reinforcement and ECCs was established. A tri-linear bond–slip constitutive model was also proposed. Ordinary steel bars exhibit low tensile strength and early yielding, which restricts an ECC’s ultra-high deformation capacity. Although fiber-woven mesh provides superior performance, its economic and construction feasibility is limited [6,7,8,9,10]. HSSWM exhibits high strength, excellent durability, and a relatively low cost. Li et al. [11,12,13,14,15,16,17,18,19,20] studied the bonding performance between high-strength steel strands and ECCs. Test results indicated that slip increases with the anchorage length and diameter of the steel strand, whereas the average bond strength decreases. Moreover, the bond strength is positively correlated with the tensile strength of ECCs and the relative cover thickness.

This study aims to address the limitations of existing bond–slip models for HSSWM–ECCs and develop a more accurate prediction model for the interfacial bonding performance. Based on Li et al. [20,21,22,23], which proposed a composite material combining HSSWM and an ECC, this study investigates the bond–slip behavior between HSSWM and an ECC through theoretical analysis and numerical simulations. The novelty of this research lies in the development of a local bond–slip model based on the free-end slip theory, using the micro-element method to achieve a more precise characterization of the bonding behavior at the interface. This model provides a theoretical foundation for accurately assessing the interfacial bonding performance.

## 2. Bonding Performance Analysis of HSSWM and an ECC

### 2.1. Overview of the Experiment

**Figure 1:**
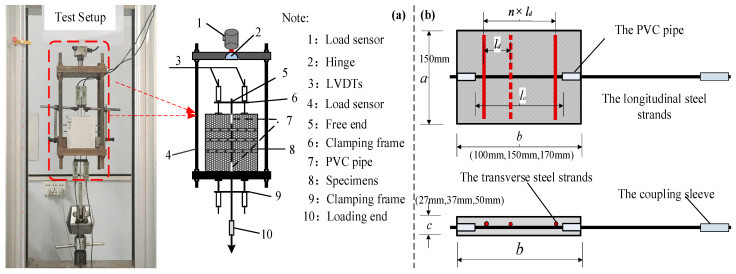
Test setup of HSSWM and the ECC. (**a**) Test loading setup; (**b**) diagram of specimens.

Based on previous experimental studies on the bond–slip behavior between HSSWM and ECCs [15], this research primarily considers factors such as the diameter of the steel strands, the spacing of transverse steel strands and the relative anchorage lengths. Unilateral pull-out tests were performed on HSSWM embedded in an ECC to investigate the influence of these factors on the bond behavior between HSSWM and ECCs. The test loading setup is shown in Figure 1.

The schematic design is shown in Figure 1. Additionally, *l_a_* refers to the effective anchorage length, and *l_d_* represents the spacing of the transverse steel strands. A specimen named H15-h2.4-30 denotes that the diameter of the relative anchorage length is 15*d*, the steel strand is 4.5 mm, and the spacing between transverse steel strands is 30 mm. The experimental results are presented in Table 1.

The effects of various parameters on the bond–slip behavior were investigated through single-sided pull-out tests conducted on 17 groups of HSSWM reinforced ECC thin plate specimens. The following conclusions were obtained through the analysis of Table 1:

(1) Two distinct failure modes were observed in the specimens during failure: steel strand pull-out and steel strand fracture.
(2) The peak bond stress between the steel strand mesh and the ECC was observed to increase as the diameter of the steel strand mesh decreased and the relative anchorage length decreased.
(3) The slip corresponding to the peak bond stress was found to increase with the decrease in the diameter of the steel strand mesh and the increase in the relative anchorage length.
(4) The inclusion of transverse steel strands exhibited a minimal impact on the peak bond stress and corresponding slip. However, the transverse steel strands effectively shared the hoop stress in the longitudinal steel strands, thereby reducing the relative slip between the steel strands and the ECC.

### 2.2. Analysis of Bond Mechanism

**Figure 2:**
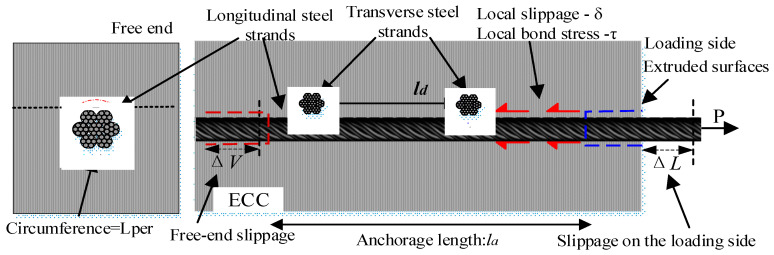
Pull model for HSSWM and ECC (*l_d_* is the spacing of the transverse steel strands).

A pull-out force model was established, as shown in Figure 2. The pull-out force *P* was applied through the strand, and a slip Δ*L* was caused at the loading end. From the equilibrium of forces, the pull-out force *P* on the steel strand was equal to the interfacial force between the steel strand and the ECC within the anchorage length. *P* was related to the bond strength between the HSSWM and the ECC. However, when the anchorage length was relatively long, the application of the average bond–slip model was unable to accurately analyze the bonding performance between the HSSWM and the ECC due to the influence of the anchoring length.

**Figure 3:**
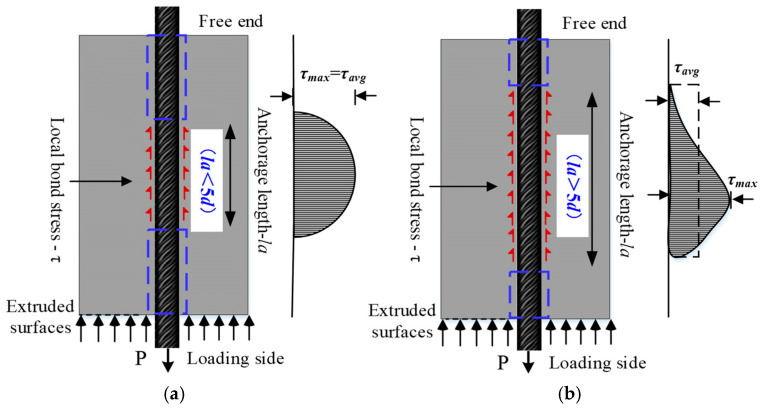
Bond stress distribution at different anchorage lengths: (**a**) short anchorage length; (**b**) long anchorage length.

When the anchorage length *l_a_* < 5*d* (where *l_a_* is the anchorage length and *d* is the diameter of the steel strands), the bond stress was distributed uniformly along the anchorage direction [24]. Conversely, when the anchorage length *l_a_* > 5*d*, the bond stress distribution was uneven. The bond stress distribution at different anchorage lengths is shown in Figure 3. Therefore, when the anchorage length of the steel strands was longer, the impact of the uneven bond stress distribution on the accurate analysis of bonding performance needed to be considered.

**Figure 4:**
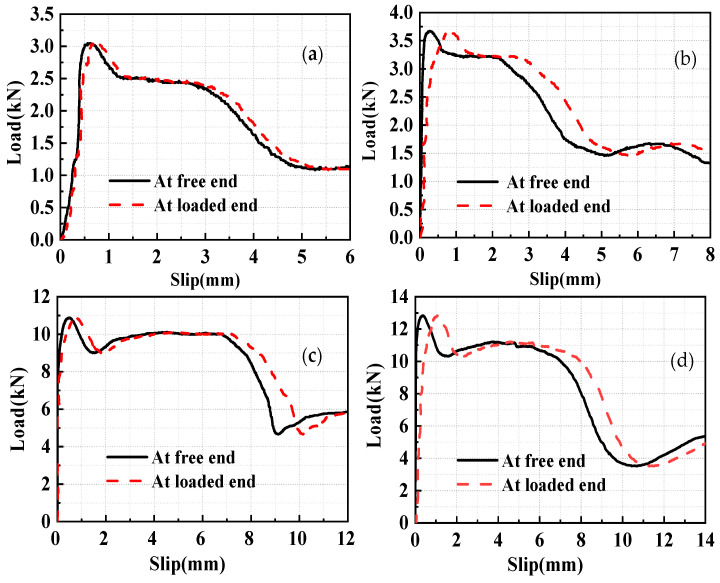
Load–slip curves at specimen ends: (**a**) H15-h2.4; (**b**) H18-h2.4; (**c**) H15-h4.5; (**d**) H18-h4.5.

Based on a previous experiment [15] on the bond–slip performance of HSSWM and an ECC, the load–slip curves are plotted in Figure 4. With other factors held constant, the differences in load–slip curves between the loading end and the free end of steel strand mesh increased progressively as the anchorage length increased. The analysis reveals that the peak pull-out load increases with the increase in anchorage length, resulting in corresponding increases in the tensile strain of the steel strands, compressive strain of the ECC, and the relative slip between the HSSWM and the ECC. When the anchorage length is short and the strain of the steel strands and the ECC is minimal, it results in a smaller relative slip between the HSSWM and the ECC.

The bond stress distribution between HSSWM and ECCs is considered a key factor in analyzing local stress characteristics and understanding bonding mechanisms. However, the existing average bond–slip model for HSSWM and ECCs has not accounted for the non-uniformity of bond stress distribution. In this study, a local bond–slip model is developed based on the average bond–slip model for HSSWM and ECCs.

## 3. Local Bond–Slip Model Between HSSWM and ECCs

### 3.1. Development of the Local Bond–Slip Model

**Figure 5:**
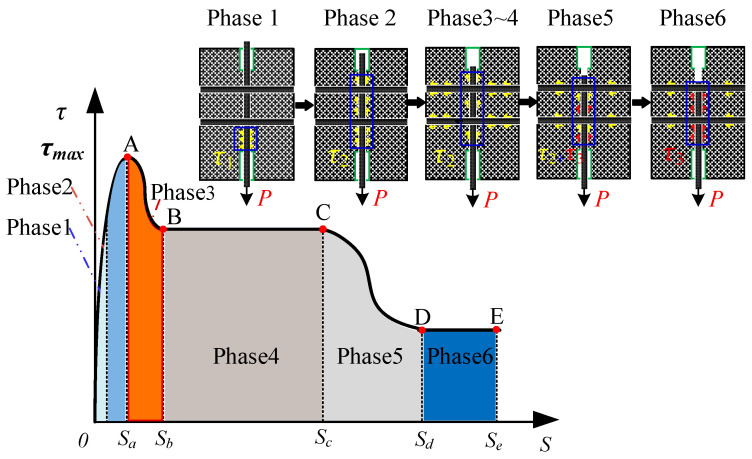
Bond–slip relationship curve between HSSWM and ECCs.

Based on the previous bonding performance tests, the average bond–slip model [25] for HSSWM and ECCs is obtained in Figure 5. The development process was divided into five phases: the ascending phase (OA), the ductile descending phase (AB), the ductile strengthening plateau (BC), the descending phase (CD), and the residual phase (DE).

The analysis indicated that the *τ*-*δ* relationship reflects the local bond characteristics [26] of the *P*-Δ response, where the relative deformation between the HSSWM and the ECC under pull-out loading was described and was applicable to any micro-segment at a local position. Based on the average bond–slip relationship model [16], the local bond–slip relationship model for the HSSWM and the ECC is proposed, as shown in Equation (1).

(1) $$\tau =\left\{\begin{array}{l} \left(1-d_{e}\right)K_{0}s\;\;\;\;\;\;\;\;\;\;\;\;\;\;\;\;0\leq s<s_{a}\\ \left[\frac{1+\varepsilon_{1}}{2}+\frac{1-\varepsilon_{1}}{2}\cos\frac{\pi \left(s-s_{a}\right)}{s_{b}-s_{a}}\right]\tau_{a}\;\;\;\;\;\;s_{a}\leq s<s_{b}\\ \varepsilon_{1}\tau_{a}\;\;\;\;\;\;\;\;\;\;\;\;\;\;\;\;\;s_{b}\leq s<s_{c}\\ \left[\frac{\varepsilon_{1}+\varepsilon_{2}}{2}+\frac{\varepsilon_{2}-\varepsilon_{2}}{2}\cos\frac{\pi \left(s-s_{c}\right)}{s_{d}-s_{c}}\right]\tau_{a}\;\;\;\;\;\;s_{c}\leq s<s_{d}\\ \varepsilon_{2}\tau_{a}\;\;\;\;\;\;\;\;\;\;\;\;\;\;\;\;\;\;\;s>s_{d} \end{array}\right.$$

where *d_e_* is the damage evolution parameter; *K*_0_ is the initial tangent stiffness of the curve; *τ_a_* (MPa) represents peak local bond stress between the HSSWM and the ECC; and *s_a_*, *s_b_*, *s_c_*, and *s_d_* (mm) represent slip values corresponding to points A, B, C, and D, respectively.

In segments BC and DE, the slope of the curve approached zero. For safety, the bond stress at the beginning of these segments was used to represent the stress across the entire segment. Thus, let *ε*_1_ = *τ_b_*/*τ_a_* and *ε*_2_ = *τ_d_*/*τ_a_*, where *ε*_1_ and *ε*_2_ are dimensionless coefficients, with *ε*_1_ representing the bond stress in segment BC and *ε*_2_ representing the bond stress in segment DE. The formulas [16] for calculating *d_e_* and *K*_0_ are as follows:

(2) $$d_{e}=1-\frac{\rho_{e}n}{n-1+{\left(s/s_{a}\right)}^{n}}$$

(3) $$K_{0}=\frac{\tau }{s}=\frac{0.8\alpha_{0}E}{d}$$

(4) $$\rho_{e}=\frac{\tau_{a}}{K_{0}s_{a}}$$

(5) $$n=\frac{K_{0}}{K_{0}s_{a}-\tau_{a}}$$

When *α*_0_ < 0.03, *α*_0_ = 0.03 (where *α*_0_ represents the instantaneous deformation depth coefficient of ECC).

### 3.2. Parameter Analysis of the Local Bond–Slip Model

In the local bond–slip relationship equation, the unknown parameters included *τ_a_*, *s_a_*, *s_b_*, *s_c_*, and *s_d_*. Other influencing factors were considered; test groups with steel strand diameters of 4.5 mm, 3.2 mm, and 2.4 mm and an anchorage length of 15*d* were selected for parameter fitting, and other test groups were used for validation.

With the reduction in anchorage length, the distribution of bond stress became more uniform. The bond stress at the interface between the steel strand mesh and the ECC was closer to the true value. The function *τ_a_* = *g*(*d*/*d*_2.4_,*f_tc_*,*l_d_*/*l_d_*_0_) was selected as the objective function, and data fitting was performed to obtain the theoretical formula for *τ_a_*, as shown in Equation (6). This formula indicates that *τ_a_* increases with increasing *f_tc_*; meanwhile, when the spacing of the transverse steel strands remains constant, a negative correlation is observed between *τ_a_* and *d*. Among them, *f_tc_* (MPa) represents the tensile strength of the ECC; *d*/*d*_2.4_ is the conversion factor for the steel strand diameter, with *d*_2.4_ = 2.4 mm; *l_d_* represents the transverse steel strand spacing, and *l_d_*/*l_d_*_0_ is the conversion factor for transverse steel strand spacing, with *l_d_*_0_ = 30 mm.

(6) $$\tau a=(2.694-0.106\frac{d}{d_{2.4}})(1.746-0.191\frac{l_{d}}{l_{d0}})\cdot f_{tc}$$

In segment BC, the pull-out load remained constant while the slip gradually increased. Simultaneously, the slope of the *τ*-*s* model approached zero, meaning *τ_b_* = *τ_c_* = *ε*_1_*τ_a_*. The bond force during this phase primarily arose from the mechanical interlock between the HSSWM and the ECC. The transverse steel strands bore part of the circumferential tensile stress in the surrounding ECC, which helped delay the loss of mechanical interlocking force. The fitting of the experimental data resulted in Equation (7). It was found that *ε*_1_ increases as the spacing of the transverse steel strands and the diameter of the steel strands decrease.

(7) $$\varepsilon 1=(1.273-0.044\frac{d}{d_{2.4}})(0.824-0.092\frac{l_{d}}{l_{d0}})$$

In segment DE, the bond force between the HSSWM and the ECC was solely provided by friction, and *τ_d_* was related to *d* and *l_d_*, where *τ_d_* = *ε*_2_*τ_a_*. The experimental data were fitted to obtain the theoretical calculation formula for *ε*_2_, as shown in Equation (8).

(8) $$\varepsilon 2=(0.845-0.044\frac{d}{d_{2.4}})(0.555-0.067\frac{l_{d}}{l_{d0}})$$

**Figure 6:**
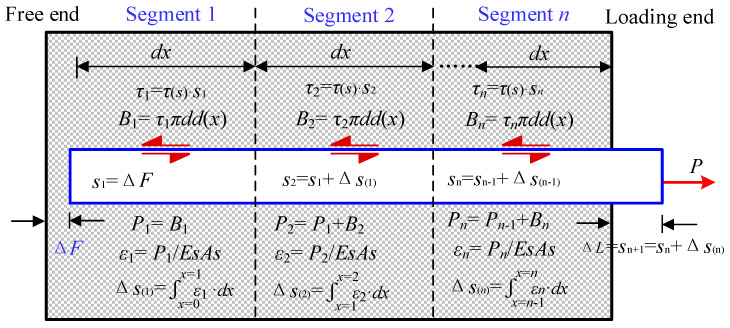
Schematic diagram of numerical analysis.

Based on the micro-element method, the steel strand mesh and ECC anchorage segment were divided into *n* sections, with each section having a micro-segment length of diameter *d*. By substituting different values of *s_a_*, *s_b_*, *s_c_*, and *s_d_*, iterative calculations were repeatedly performed and compared with the experimental results. The specific iterative calculation process is illustrated in Figure 6.

Based on the calculation process in Figure 6, the slip and bond force for each micro-segment within the anchorage length was obtained, allowing the theoretical pull-out force *P_n_* to be calculated. When the error between *P_n_* and the experimental pull-out force *P_t_* was less than 5%, the initially selected parameters were considered characteristic parameters. Otherwise, the initial parameters needed to be reset, and iterative calculations were performed until the error fell within the acceptable range. After the characteristic parameters of the local bond–slip relationship model were determined, the bond forces and slip amounts at each point within the anchorage length range could be obtained through iterative calculations.

### 3.3. Validation of the Local Bond–Slip Model

The test group with an anchorage length of 15*d* was selected as the fitting group, while the other test groups were used as validation groups for iterative analysis. The theoretical calculation results were compared with the experimental results and the average bond–slip model results to verify the applicability and accuracy of the local bond–slip model.

**Figure 7:**
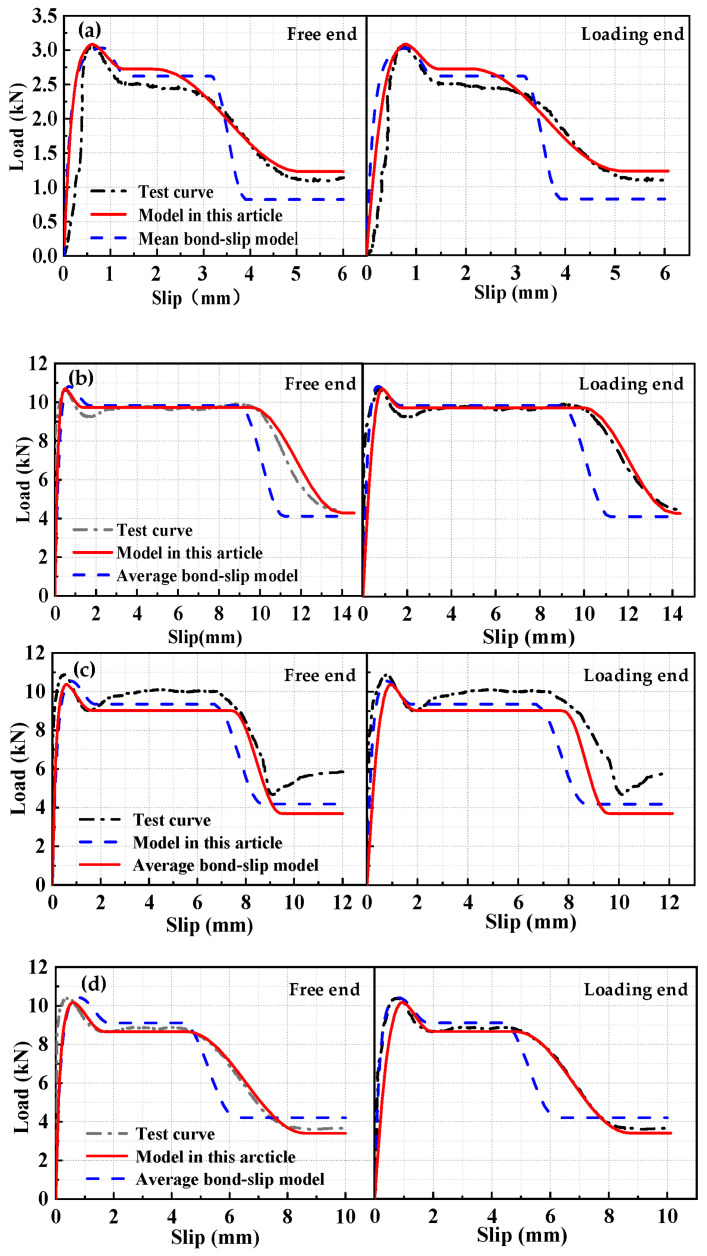
Comparison of load-slip curves for the fitting group: (**a**) H15-h2.4-30; (**b**) H15-h4.5-20; (**c**) H15-h4.5-30; (**d**) H15-h4.5-40.

**Figure 8:**
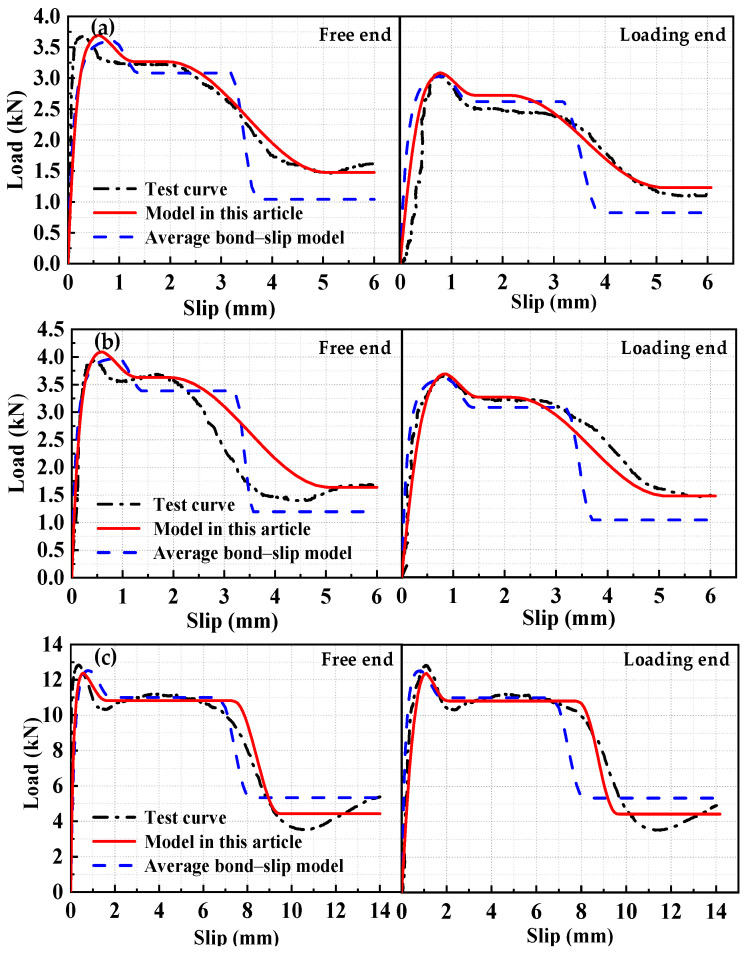

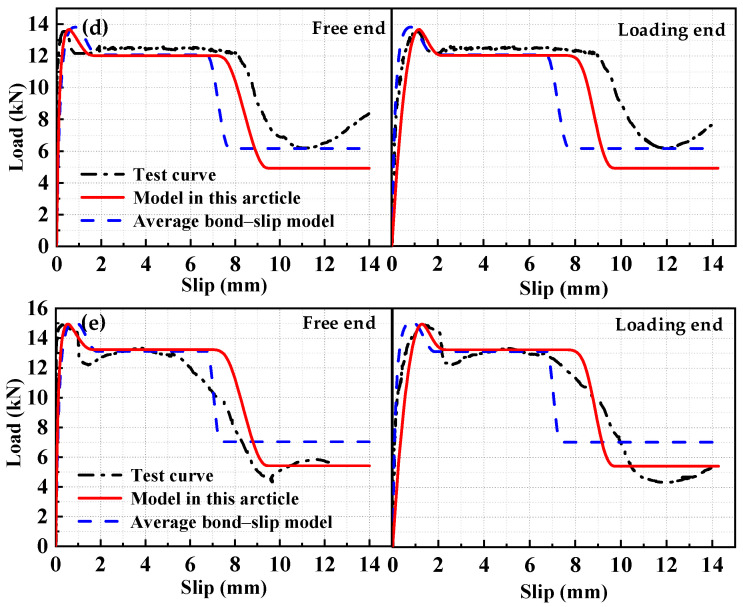
Comparison of load–slip curves for the validation group: (**a**) H18-h2.4-30; (**b**) H20-h2.4-30; (**c**) H18-h4.5-30; (**d**) H20-h4.5-30; (**e**) H20-h4.5-30.

Two sets of specimens were analyzed based on the local and average bond–slip models to obtain the load–slip curves for the free end and the loading end, as shown in Figure 7 and Figure 8.

The calculated results of the local bond–slip model were in good agreement with the experimental results. The local bond–slip model for the HSSWM and the ECC provided a more accurate representation of the bond–slip relationship compared with the average bond–slip model.

## 4. Numerical Analysis

### 4.1. Numerical Simulation Validation of the Local Bond–Slip Relationship Between the HSSWM and the ECC

**Figure 9:**
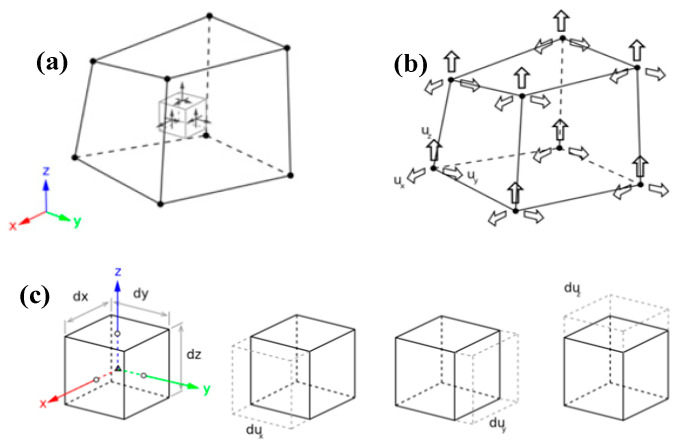
The HX24L element of the ECC. (**a**) HX24L element of the ECC; (**b**) translational displacements along the x, y, and z directions at each node; (**c**) deformations generated by displacements in different directions at each node.

**Figure 10:**
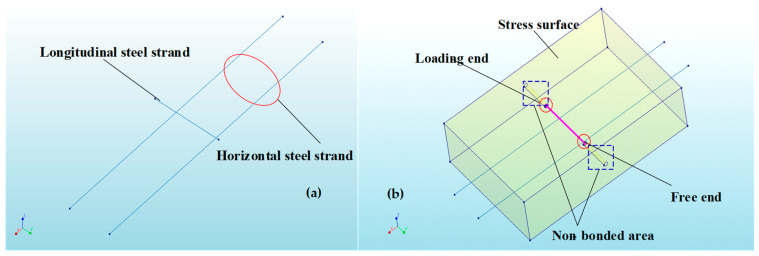
The 3D nonlinear FE model: (**a**) HSSWM model; (**b**) component model.

**Figure 11:**
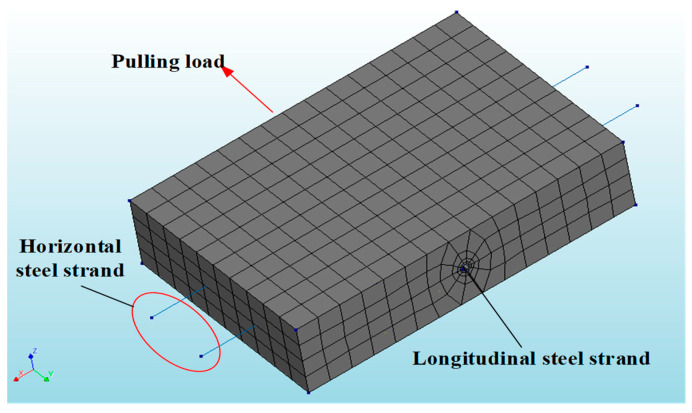
Mesh division of 3D nonlinear FE model.

**Figure 12:**
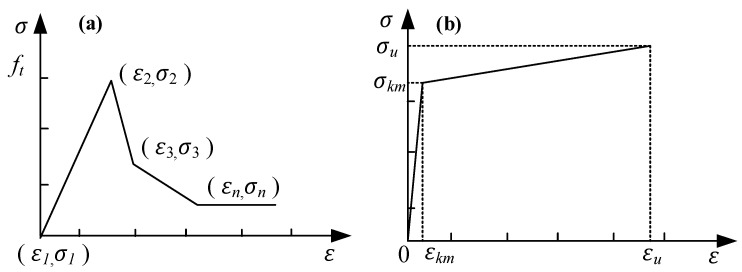
Tensile constitutive model of the ECC. (**a**) Based on the total strain multi-segment linear tensile softening model; (**b**) tensile stress–strain curve for the ECC.

A pull-out test model was established using the FE software DIANA (2017) to validate the accuracy of the proposed model. In the 3D nonlinear FE model, the ECC was modeled with eight-node (HX24L) solid elements from the software package, as shown in Figure 9. Embedded elements were used for the steel strands, allowing the strands to automatically embed into the ECC and form a cohesive unit with the surrounding elements. The unbonded segment was created by introducing holes in the ECC, ensuring that the model’s loading conditions matched those of the test. In the non-bonded segment, holes were drilled in the ECC to ensure that the loading conditions in the model were consistent with those in the experiments. Schematic diagrams of the FE model are shown in Figure 10 and Figure 11. The tensile stress–strain behavior [27] of the ECC was defined according to the constitutive model in Equation (9), and the tensile constitutive model of the ECC is shown in Figure 12.

(9) $$\sigma =\left\{\begin{array}{l} E_{t}\varepsilon \;\;\;\;\;\;\;\;\varepsilon \leq \varepsilon_{km}\\ (c\frac{\varepsilon }{\varepsilon_{u}}+1-c)\sigma_{u}\;\;\;\varepsilon_{km}<\varepsilon \leq \varepsilon_{u} \end{array}\right.$$

In the equation, *σ_km_* (MPa) represents the nominal cracking stress of the ECC, *ε_km_* is the nominal cracking strain of the ECC, *σ_u_* (MPa) is the peak tensile stress of the ECC; *ε_u_* is the peak tensile strain of the ECC; *E_t_* is the elastic modulus of the ECC. Parameter values based on the experimental results are shown in Table 2.

(10) $$\sigma =\left\{\begin{array}{l} \left[1.1\frac{\varepsilon }{\varepsilon_{u}}+0.5{\left(\frac{\varepsilon }{\varepsilon_{u}}\right)}^{5}-0.6{\left(\frac{\varepsilon }{\varepsilon_{u}}\right)}^{6}\right]\sigma_{u}\;\;\;\;0\leq \frac{\varepsilon }{\varepsilon_{u}}<1\\ (\frac{0.15{\left(\frac{\varepsilon }{\varepsilon_{u}}\right)}^{2}}{1-2\frac{\varepsilon }{\varepsilon_{u}}+1.15{\left(\frac{\varepsilon }{\varepsilon_{u}}\right)}^{2}})\sigma_{u}\;\;\;\;\;\;\;\frac{\varepsilon }{\varepsilon_{u}}>1 \end{array}\right.$$

**Figure 13:**
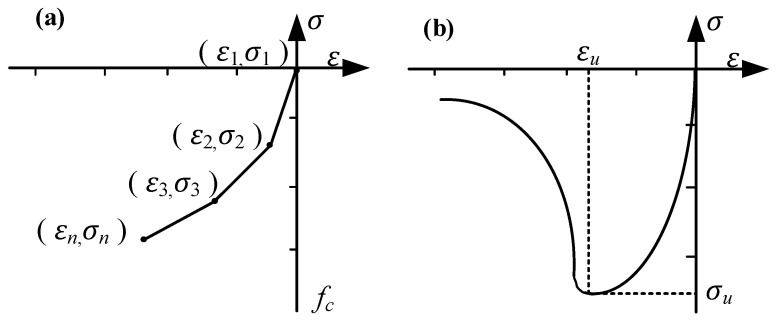
Compressive constitutive model of the ECC. (**a**) Based on the total strain multi-segment linear compression model; (**b**) compressive stress–strain relationship curve of the ECC.

The compressive constitutive model of the ECC is shown in Figure 13. The compressive constitutive relation of the ECC is expressed in Equation (10). In the equation, *σ* and *ε* represent the compressive stress and strain of the ECC, respectively, with *σ_u_* = 32.45 MPa and *ε_u_* = 0.22%.

**Figure 14:**
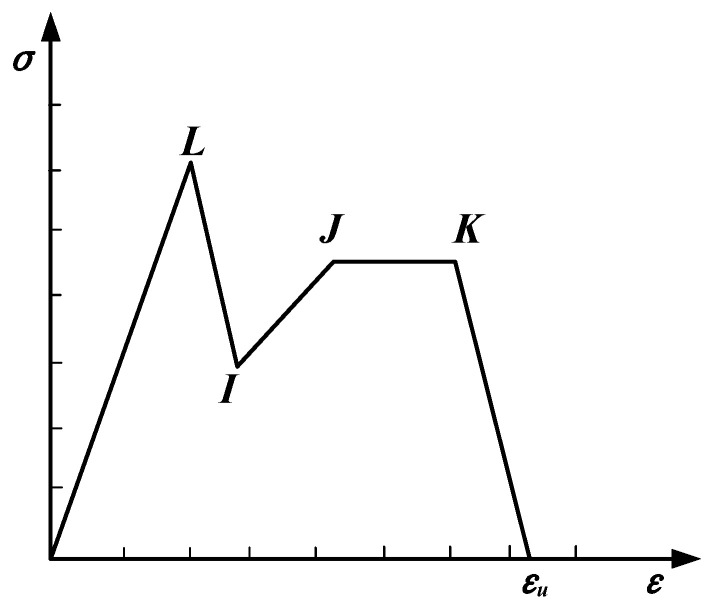
Fiber concrete model.

The fiber concrete model provided by DIANA was employed in this study, as shown in Figure 14. In the model, point *L* corresponds to the cracking stress of the material, and point *K* represents the ultimate stress. Points *I* and *J* are used to interpolate the values between the cracking stress and the ultimate stress. The experimentally measured cracking stress, along with the values for the strain hardening and softening stages were input into the model. This constitutive model was then adjusted to include the elastic stage, the strain hardening stage, and the strain softening stage.

**Figure 15:**
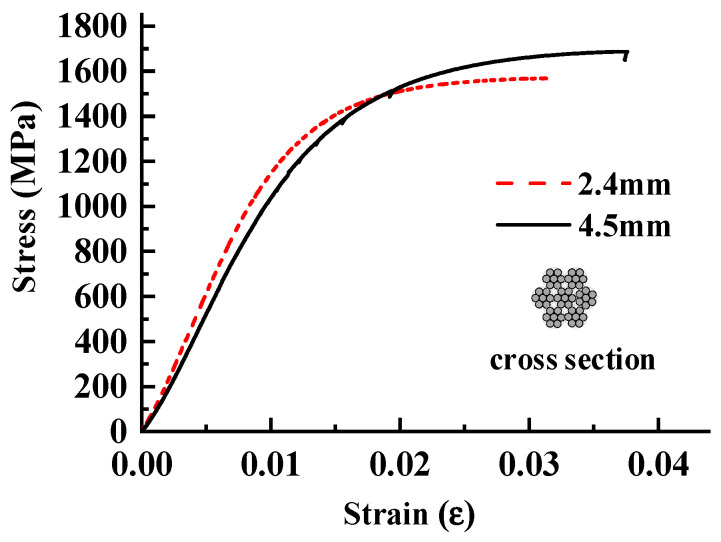
Tensile stress–strain curves of steel strands with different diameters.

The constitutive model for the HSSWM adopted the Von Mises plastic strain–yield stress model. The tensile stress–strain curves for steel strands of different dimeters obtained from experiments are shown in Figure 15. The constitutive relationship for HSSWM is expressed in Equation (11).

(11) $$y=Ax+Bx^{2}+Cx^{3}$$

where *x* = *ε*/*ε_su_*, y = *σ*/*σ_su_*, *ε_su_* represents the tensile strain of the steel strand, and *σ_su_* (MPa) is the tensile strength of the steel strand. Parameters *A*, *B*, and *C* were determined through experimentation. The parameters for steel strands of different diameters are listed in Table 3.

**Figure 16:**
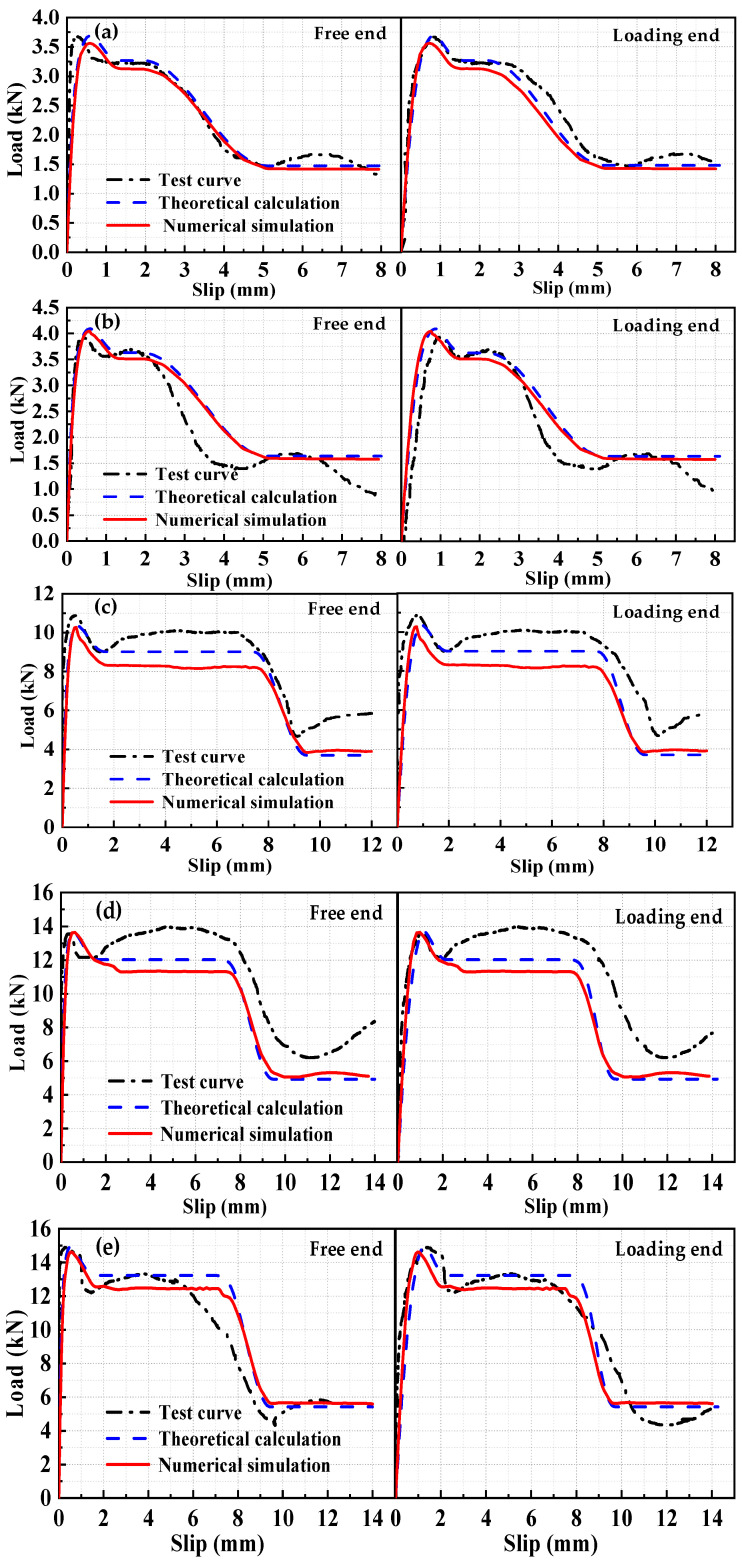
Load–slip curve: (**a**) H18-h2.4-30; (**b**) H20-h2.4-30; (**c**) H15-h4.5-30; (**d**) H20-h4.5-30; (**e**) H22-h4.5-30.

Displacement control was utilized to apply the load to the end of the longitudinal steel strands at the loading end, as shown in Figure 11. To simulate the constraints of the pull-out test, the loading face of the ECC at the loading end was fully restrained to ensure consistency with the experimental conditions (where the loading face is limited in displacement by a steel plate). Constraints were applied at the loading point (end of the longitudinal steel strand), with only the loading direction left unrestrained. The numerical results of the 3D nonlinear FE model are presented in Table 4 and Figure 16.

Table 4 and Figure 16 demonstrate that the theoretical, numerical simulation, and experimental curves exhibit minimal deviation. The numerical simulation curves for the loading end and free end closely aligned with the theoretical calculation curves in the OA, CD, and DE segments. However, the fitting effect of the BC curve was unsatisfactory, particularly for specimens with a diameter of 4.5 mm. The reason for this discrepancy was that ribs were formed on the surface of the steel strands during winding, which enhanced the interaction between the HSSWM and the ECC. The steel strand was not finely processed in the FE simulation. The reduction in frictional force between the HSSWM and the ECC led to a more pronounced decrease in load. The experimental curves indicate that in the BC segment, there is a trend of load recovery after an initial drop. This load reduction is attributed to slippage in regions with high compressive stress, where bond deterioration occurs due to local stress exceeding adhesion limits. Conversely, in areas with lower compressive stress, the load tends to increase as bond stability is more sustained. Consequently, the load–slip curve shows an upward trend in the ductile phase, as increased slip promotes progressive load redistribution and delayed failure.

### 4.2. Numerical Simulation Analysis of the Bond–Slip Performance Between the HSSWM and the ECC

To further investigate the bond behavior between the HSSWM and the ECC, extended parameter analysis was conducted. An FE model was established using DIANA to investigate the influence of various parameters on the local bonding mechanical properties. To explore the impact of ECC tensile strength on the local bond–slip model, a single-factor extended analysis was performed. Table 5 presents the simulated material parameters corresponding to various ECC tensile strengths, as reported in [28].

Based on the previously established model (H15-h2.4/4.5-30), two diameters of 3.2 mm and 3.6 mm were selected to investigate the influence of strand diameter on bonding performance. The parameters of the simulated specimens are shown in Table 6, and the mechanical properties of the steel strands are presented in Table 7.

The arrangement and spacing of transverse steel strands affect the bond and anchorage performance between the HSSWM and the ECC. To investigate the impact of transverse steel strand spacing on the model, different spacings were analyzed. The parameters of the simulated specimens were shown in Table 8.

**Figure 17:**
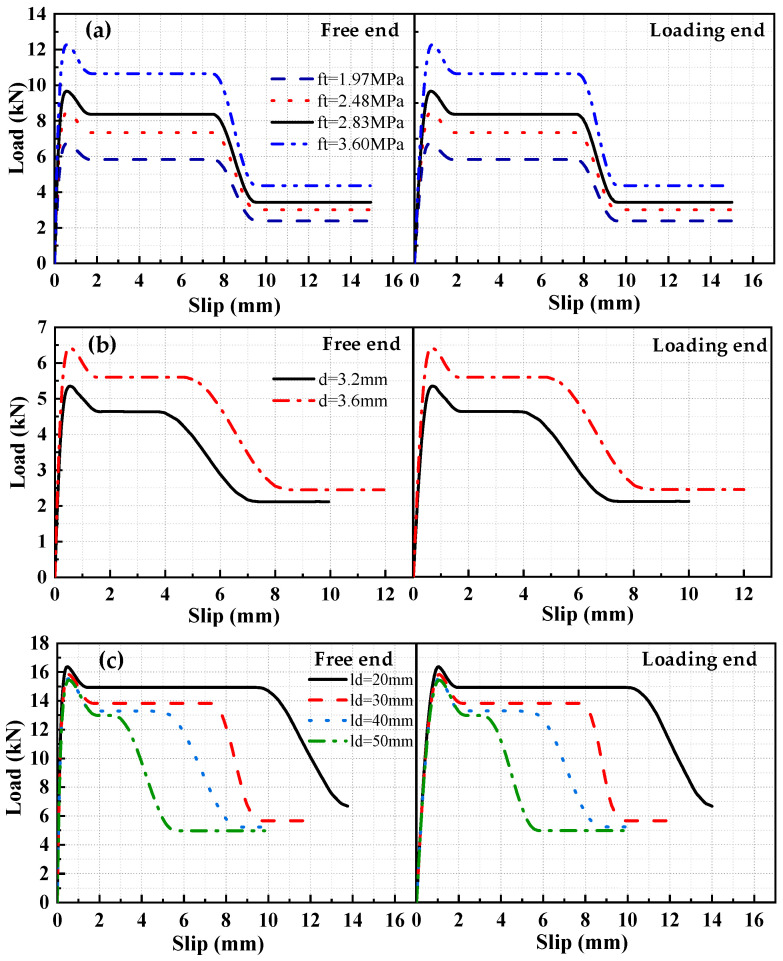
Load displacement curve under different conditions.

The load–slip curves of specimens with different ECC tensile strengths were obtained by FEA, as shown in Figure 17a. The figure displays that ECC tensile strength had significant influence on the load–slip curve shape. The shape of the entire curve improved with the increase in tensile strength. The load values at various characteristic points of the curve increased, but the corresponding slip values showed little variation. The load–slip curves for specimens with different steel strand diameters is shown in Figure 17b. The figure illustrates that the pulling force of the specimen increases with the diameter of the steel strands. This was attributed to the positive correlation between the pulling force *F* and the diameter *d* during the microelement calculation process, as shown in Equation (12). Figure 17c illustrates the load–slip curves for specimens with different transverse spacing of steel strands. The figure displays that the impact of varying transverse spacing on the peak load is minimal. As the spacing decreased, the load at each stage increased, and the length of the ductile horizontal segment became longer. This further demonstrated that the spacing of transverse steel strands significantly impacts the local bond–slip relationship model. The reason is that the transverse steel strands partially bear the tensile stress transferred from the surrounding longitudinal steel strands within the ECC. The resistance to cracking within ECC enhanced the ductility of the specimen during failure while delaying the loss of mechanical interlocking force. The number of steel strands increased as the spacing decreased, resulting in a more pronounced delay effect.

(12) $$F=\sum_{i=1}^{n}\tau_{i}\pi dl_{i}$$

**Figure 18:**
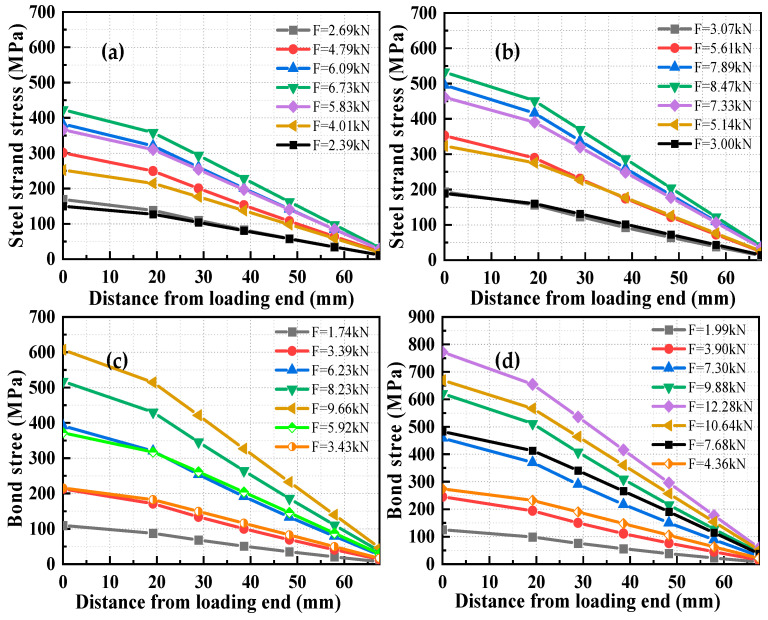
Stress distribution of longitudinal steel strand of specimens with different ECC tensile strengths: (**a**) *f_tc_* = 1.97 MPa; (**b**) *f_tc_* = 2.48 MPa; (**c**) *f_tc_* = 2.83 MP; (**d**) *f_tc_* = 3.60 MPa.

The stress distribution of longitudinal steel strands under different ECC tensile strength is shown in Figure 18. It can be observed that before reaching the peak load, the stress in the steel strands increased with the applied load. When the peak load was reached, the stress at various positions along the longitudinal steel strands decreased as the load was reduced. The stress in the steel strands decreased from the loading end to the free end at the same load level.

**Figure 19:**
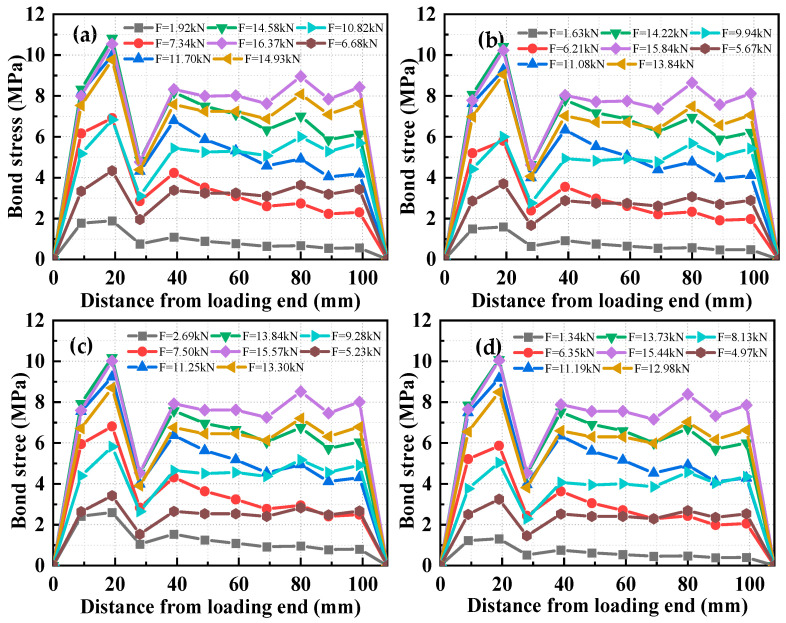
Distribution curve of along the anchorage length direction. (**a**) *l_d_* = 20 mm; (**b**) *l_d_* = 30 mm; (**c**) *l_d_* = 20 mm; (**d**) *l_d_* = 30 mm.

The distribution curve of bond stress between the HSSWM and the ECC along the anchorage length is shown in Figure 19. The figure displayed that the maximum value occurs near the loading end at a distance of 20 mm, while the minimum value is located close to the free end. The maximum bond stress did not migrate toward the free end as load levels increased. The analysis indicated [29] that when *d* = 4.5 mm, the critical anchorage length was 25*d*, which is close to the critical anchorage length. The pulling force applied to the longitudinal steel strands at the loading end was challenging to transmit to the free end, leading to lower bond stresses near the free end. A sharp drop in bond stress was observed at 30 mm from the loading end. The analysis indicates uneven node distribution during FE simulation. When nodes along the longitudinal steel strands were selected for analysis, the nodes shifted from one side of the transverse steel strand to the other during the pull-out process. The constraining effect of the transverse steel strands caused minimal variation in the stress of the rebar at these two nodes, resulting in a reduction in bond stress.

The local bond–slip relationship model between the HSSWM and the ECC proposed in this paper was valid for the range 20 mm ≤ *l_d_* ≤ 60 mm due to the limitations of the experimental study. The local bonding performance at other spacings required further investigation.

## 5. Analytical Model of Bonding Performance at the Interface Between the HSSWM and the ECC

### 5.1. Bond Capacity Prediction Model

**Figure 20:**
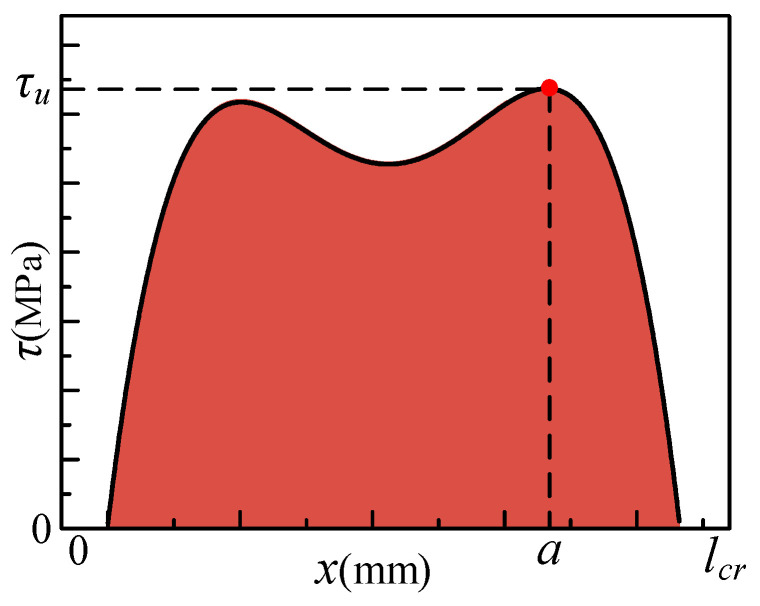
Bond stress distribution curve along the anchorage length.

Based on the application of the micro-element method (detailed in Section 3.2) and the local bond–slip model between the HSSWM and ECC, the bond stress distribution along the anchorage length was obtained using FE software DIANA (2017), as shown in Figure 20 and Table 9.

The maximum bond stress was located near the free end at the peak load. A fitting analysis of pull-out specimens with different anchorage lengths was conducted, resulting in the following function to describe the variation in bond stress with anchorage position:

(13) $$\tau (x)=A+B_{1}x+B_{2}x^{2}+B_{3}x^{3}+B_{4}x^{4}$$

where *A*, *B*_1_, *B*_2_, *B*_3_, and *B*_4_ are fitting coefficients. The fitting results and corresponding coefficients are presented in Table 10.

An integral over the anchorage length of the component was performed to calculate the bond capacity prediction model for the HSSWM and the ECC within the anchorage length, as shown in Equation (14).

(14) $$P_{u}=\int_{0}^{l}\pi d(A+B_{1}x+B_{2}x^{2}+B_{3}x^{3}+B_{4}x^{4})dx$$

In the equation, *P_u_* (kN) is the bond capacity; *l_cr_* (mm) is the critical anchorage length, with values provided in Section 4.2. The bond capacities of the HSSWM and the ECC for each specimen were calculated using Equation (14) and compared with the results from numerical analysis and experimental data, as shown in Table 10.

Table 11 displays that the average ratio of the predicted bond capacity model results to the experimental results is 0.916, with a standard deviation of 0.032 and a coefficient of variation of 0.035. The predicted model results aligned well with the experimental results overall. The proposed bond load-bearing capacity model effectively predicted the bond load-bearing capacity between the HSSWM and the ECC.

### 5.2. Critical Anchorage Length Prediction Model

The bond strength *P_u_* between the HSSWM and the ECC at the critical anchorage length *l_cr_* can be calculated from the bond stress *τ*, as shown in Equation (12). The critical anchorage length of the longitudinal steel strand is determined using the microelement method and the local bond–slip model. The anchorage segment is divided into multiple micro-segments, and iterative calculations are performed until the error between the rupture force of the steel strand and the theoretical value is less than 5%, thereby determining the critical anchorage length. The results of the iterative calculations are analyzed, and the findings are presented in Table 12.

Table 12 displays that the error between the iterative pull-out force and the rupture force of the longitudinal steel strands for each specimen is less than 1%, satisfying the required accuracy. The anchorage length calculated using the local bond–slip model can be considered the critical anchorage length of the longitudinal high-strength steel strands in the ECC.

**Figure 21:**
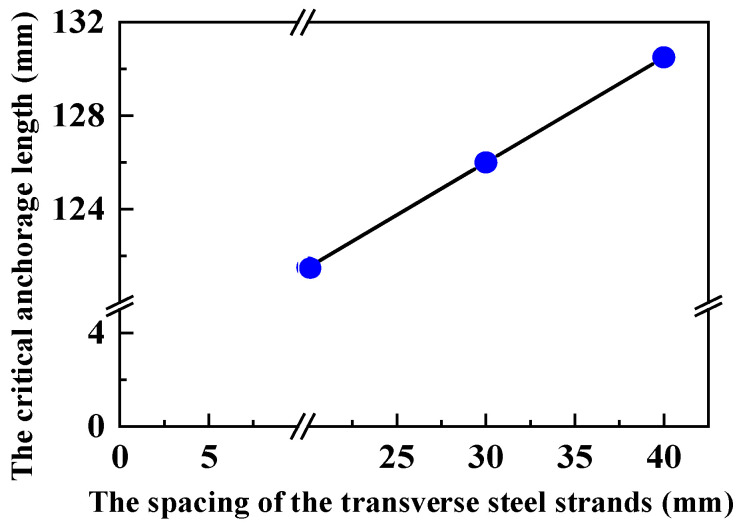
Effect of the spacing between transverse steel tendons on the critical anchorage length.

Experimental studies show that the critical anchorage length of the longitudinal steel strands in ECCs is positively linearly correlated with the spacing of the transverse steel strands. Specifically, the critical anchorage length *l_cr_* increases as the spacing *l_d_* of the transverse steel strands increases, as shown in Figure 21.

A fitting process was performed with the target function *l_cr_* = *f*(*d*/*d*_2.4_,*l_d_*,*f_y_*,*f_tc_*). The critical anchorage length prediction expression (15) is obtained, with *R*^2^ = 0.936.

(15) $$l_{cr}=0.332\frac{f_{y}}{f_{t}}d(0.107+0.013\frac{l_{d}}{l_{d0}})$$

In the equation, *f_y_* is the design tensile strength of the steel strands, with *γ*_s_ = 1.2. When *d* = 2.4 mm, *f_y_* = 1307 MPa, and when *d* = 4.5 mm, *f_y_* = 1406 MPa. *f_tc_* is the design tensile strength of the ECC, with [29] *γ*_c_ = 1.4, and the calculated value of *f_tc_* is 2.02 MPa.

The critical anchorage length calculated by Equation (15) ensured structural safety. From the comparative analysis in Table 13, it can be observed that the critical anchorage length prediction model proposed in this paper yields more accurate results than those reported in the literature.

## 6. Conclusions

The following conclusions were obtained:

(1) A local bond–slip model was proposed based on the average bond–slip model for HSSWM and ECCs. The local bond–slip model provides a more accurate analysis of the bonding performance between HSSWM and ECCs compared to the average bond–slip model.
(2) The established local bond–slip relationship model was validated through numerical simulation. The results indicated that the theoretical calculation curve, numerical simulation curve, and experimental curve were found to be in good agreement. The FE modeling approach used can effectively simulate the bond mechanical behavior between HSSWM and ECCs.
(3) The effects of ECC tensile strength, steel strand diameter, and transverse strand spacing on local bond–slip mechanical behavior were investigated through finite element analysis. The results indicated that the stress and bond stress of the longitudinal steel strands were slightly reduced as the diameter increased. The arrangement of transverse steel strands had a significant impact on the ductility of bond failure, increasing as the steel strand spacing decreased. The stress and bond stress of the longitudinal steel strands at the loading end were found to increase with the enhancement of the ECC tensile strength when the peak load was reached.
(4) Based on the local bond–slip model developed in this paper, the micro-element method was used to analyze the interfacial bonding performance between the HSSWM and an ECC. A prediction model for the critical anchorage length and bond capacity of the HSSWM in ECCs was proposed. Comparative analysis shows that the theoretical calculations are in good agreement with the experimental results, confirming the accuracy of the model.

Future research could focus on the bond mechanical properties of HSSWM and ECCs under cyclic loading, further exploring their stress characteristics and failure modes in practical engineering applications. In addition, durability and environmental effects should also be key areas of focus for future investigations.

## Figures and Tables

**Table 1:** Test results.

Specimen Numbering	Size/mm	*T_a_*	*τ_a_*	*s_a_*	Results
H15-h4.5-0	150 × 150 × 50	10.92	11.32	0.92	Pull-Out
H15-h4.5-20	150 × 150 × 50	11.14	11.68	0.82	Pull-Out
H15-h4.5-30	150 × 150 × 50	11.10	11.14	0.72	Pull-Out
H15-h4.5-40	150 × 150 × 50	10.38	10.89	0.76	Pull-Out
H18-h4.5-30	150 × 150 × 50	12.11	11.37	0.75	Pull-Out
H20-h4.5-30	150 × 150 × 50	12.81	10.07	1.39	Pull-Out
H22-h4.5-30	150 × 150 × 50	14.11	10.09	0.82	Pull-Out
H25-h4.5-30	150 × 170 × 50	14.93	9.44	0.98	Pull-Out
H28-h4.5-30	150 × 170 × 50	16.18	9.09	—	Fracture
H15-h3.2-30	150 × 100 × 37	5.49	11.38	0.78	Pull-Out
H18-h3.2-30	150 × 100 × 37	6.47	11.17	0.84	Pull-Out
H20-h3.2-30	150 × 100 × 37	7.08	11.11	0.93	Pull-Out
H22-h3.2-30	150 × 100 × 37	7.56	10.75	—	Fracture
H15-h2.4-30	150 × 100 × 27	3.19	11.72	1.11	Pull-Out
H18-h2.4-30	150 × 100 × 27	3.73	11.25	0.87	Pull-Out
H20-h2.4-30	150 × 100 × 27	3.94	10.97	0.91	Pull-Out
H22-h2.4-30	150 × 100 × 27	4.37	10.94	—	Fracture

Note: *T_a_* (kN) represents the peak pull-out load. *τ_a_* (MPa) denotes the average bond stress corresponding to the peak pull-out load. *s_a_* (mm) refers to the average slip displacement at the peak pull-out load.

**Table 2:** Tensile characteristic parameters of the ECC.

*σ_km_*/MPa	*ε_km_*/%	*σ_u_*/MPa	*ε_u_*/%	*E_t_*/GPa	*c*	*σ_km_*/MPa
1.89	0.014	2.83	2.2	14.5	0.31	1.89

**Table 3:** Steel strand parameters.

*d*/mm	*σ_su_*/MPa	*E_s_*/GPa	*ε_su_*/%	*A*	*B*	*C*
2.4	15,683.30	130	3.07	3.33	−3.66	1.33
4.5	1687.45	108	3.78	2.90	−2.80	0.90

**Table 4:** Error analysis of characteristic parameter values at the loading and free ends for the validation group.

Site	Parm	*P_a_*		*s_a_*		*P_b_*		*s_b_*	
		LC	FEM	LC	FEM	LC	FEM	LC	FEM
L	μ	1.011	0.991	1.025	0.830	1.028	1.000	1.069	0.898
F		1.011	0.991	1.508	1.508	1.028	1.000	1.591	1.438
L	Cv	0.020	0.030	0.117	0.075	0.041	0.032	0.119	0.142
F		0.020	0.030	0.256	0.256	0.041	0.032	0.235	0.237
L	ERR	1.14	0.87	2.48	17.05	2.81	0.01	6.89	10.23
F		1.54	6.98	12.81	9.14	4.97	6.74	4.03	4.91
L	μ	0.985	0.930	0.872	0.909	1.050	1.067	0.960	0.951
F		0.985	0.930	0.967	1.053	1.050	1.067	1.040	1.039
L	Cv	0.070	0.058	0.144	0.103	0.157	0.169	0.166	0.144
F		0.070	0.058	0.152	0.125	0.157	0.169	0.153	0.128
L	ERR	1.14	0.87	50.81	50.81	2.81	0.01	59.11	43.76
F		1.54	6.98	3.35	5.35	4.97	6.74	4.01	3.93

Note: L and F represent the slip positions at the loading end and free end, respectively.

**Table 5:** Parameters of simulated ECC specimens.

Number	*σ_cu_*/MPa	*σ_tcr_*/MPa	*σ_tu_*/MPa	*ε_cu_*/%	*ε_tcr_*/%	*ε_tu_*/%	*E_t_*/Gpa	*μ*
32.64	1.97	1.97	0.22	0.027	0.027	13.7	0.26	32.64
33.30	1.92	2.48	0.54	0.035	0.44	14.5	0.26	33.30
32.30	2.45	3.60	0.45	0.043	1.35	14.5	0.26	32.30

Note: *σ_cu_* represents the compressive strength of the ECC; *σ_tcr_* is the cracking strength of the ECC; *σ_tu_* is the tensile strength of the ECC; *ε_cu_* is the peak compressive strain of the ECC; *ε_tcr_* is the cracking strain of the ECC; *ε_tu_* is the peak tensile strain of the ECC; *μ* is the Poisson’s ratio of the ECC.

**Table 6:** Parameters of simulated steel strand specimens.

Number	*σ_tu_*/MPa	Size/mm	*d*/mm	*l_a_*/mm	*l_d_*/mm
1	2.83	150 × 100 × 37	3.2	15d	30
2	2.83	150 × 100 × 37	3.6	15d	30

**Table 7:** Mechanical properties of steel strands.

Number	*d*/mm	*A_s_/*mm^2^	*σ_su_*/MPa	*E_s_*/GPa	*ε_su_*/%
1	3.2	4.94	1589	97	4.08
2	3.6	6.16	1620	140	-

Note: *A_s_* represents the measured cross-sectional area of the steel strand; *E_s_* is the elastic modulus of the steel strand.

**Table 8:** Parameters of simulated specimens.

Number	Size/mm	*σ_tu_*/MPa	*l_a_*/mm	*l_d_*/mm	*d*/mm
1	150 × 150 × 50	2.83	24*d*	20	4.5
2	150 × 150 × 50	2.83	24*d*	30	4.5
3	150 × 150 × 50	2.83	24*d*	40	4.5
5	150 × 150 × 50	2.83	24*d*	50	4.5

**Table 9:** Specimen piece H15-h4.5-30 bond stress at different anchoring positions under the same load.

	*P* (kN)	1.74	3.39	6.23	8.23	9.33	9.66
*x* (mm)		*τ*_1_	*τ*_2_	*τ*_3_	*τ*_4_	*τ*_5_	*τ_u_*
0		0	0	0	0	0	0
10		2.5702	4.8923	8.3973	10.2339	10.8780	10.8435
19		2.2417	4.3245	7.6838	9.7917	10.6814	10.8133
29		2.0137	3.9309	7.2266	9.4727	10.6648	10.9555
39		1.8172	3.5708	6.7190	9.1082	10.4798	10.9304
48		1.6879	3.3337	6.3842	8.8212	10.3642	10.9223
58		1.6121	3.1939	6.1813	8.6377	10.2391	10.8896
68		0	0	0	0	0	0

Note: *x* is the distance from the loading end; *P* is the pull-out load.

**Table 10:** Fractional coefficients.

*f_t_*/MPa	*d*/mm	*l_d_*/mm	*l_a_*/*d*	*A*	*B*_1_	*B*_2_	*B*_3_	*B*_4_	*R* ^2^
2.83	4.5	30	15	0.090	1.871	−0.103	0.002	−1.65 × 10^−5^	0.974
2.83	4.5	30	18	−0.107	1.664	−0.078	0.001	−8.84 × 10^−6^	0.938
2.83	4.5	30	20	0.293	1.452	−0.061	0.001	−5.61 × 10^−6^	0.938
2.83	4.5	30	22	0.179	1.331	−0.051	7.56 × 10^−4^	−3.81 × 10^−6^	0.933
2.83	2.4	30	15	0.200	3.667	−0.376	0.015	−2.11 × 10^−4^	0.942
2.83	2.4	30	18	0.162	3.164	−0.279	0.010	−1.11 × 10^−4^	0.961
2.83	2.4	30	20	1.054	2.823	−0.230	0.007	−7.55 × 10^−5^	0.820
2.83	4.5	20	15	0.095	1.940	−0.107	0.002	−1.72 × 10^−5^	0.974
2.83	4.5	40	15	0.867	1.841	−0.101	0.002	−1.63 × 10^−5^	0.975

**Table 11:** Comparison of calculated bond capacity values.

Number	*P^p^_u_*/kN	*P^t^_u_*/kN	*P^p^_u_*/*P^t^_u_*
H15-h4.5-20	9.63	10.66	0.903
H15-h4.5-30	9.31	10.87	0.856
H15-h4.5-40	9.16	10.38	0.882
H18-h4.5-30	11.52	12.48	0.923
H20-h4.5-30	12.58	13.55	0.928
H22-h4.5-30	13.84	14.89	0.929
H15-h2.4-30	2.81	3.05	0.922
H18-h2.4-30	3.37	3.60	0.917
H20-h2.4-30	3.83	3.97	0.978

Note: *P^p^_u_* is the calculated value from the bond capacity prediction expression; *P^t^_u_* is the experimental bond capacity value.

**Table 12:** Results of the iterative calculations.

Number	*f_t_*/MPa	*d*/mm	*l_d_*/mm	*l*^1^*_cr_/*mm	*P_su_*/kN	*P_cu_*/kN	*P_su_*/*P_cu_*
1	2.02	4.5	20	27*d*	13.52	13.53	1.00
2	2.02	4.5	30	28*d*	13.52	13.49	1.00
3	2.02	4.5	40	29*d*	13.52	13.60	0.99
4	2.02	2.4	30	25*d*	3.68	3.64	1.01

**Table 13:** Comparison of critical anchorage lengths.

Number	*l*^t^*_cr_*/mm	*L*^30^*_cr_*/mm	*L*^31^*_cr_*/mm	*l*^2^*_cr_*/mm
H20-h4.5-2.02	25*d*	19*d*	25*d*	27*d*
H30-h4.5-2.02	25*d*	19*d*	25*d*	28*d*
H40-h4.5-2.02	25*d*	19*d*	25*d*	29*d*
H30-h2.4-2.02	22*d*	18*d*	23*d*	25*d*

Note: *l_tcr_* is the experimental value; *l*^30^*_cr_* is the calculated value from reference [30]; *l*^31^*cr* is the calculated value from reference [31]; *l*^2^*_cr_* is the calculated value from Equation (15).

## Data Availability

The original contributions presented in this study are included in the article. Further inquiries can be directed to the corresponding author.
